# Factors associated with negative T-SPOT.TB results among smear-negative tuberculosis patients in China

**DOI:** 10.1038/s41598-018-22495-3

**Published:** 2018-03-09

**Authors:** Wanli Kang, Meiying Wu, Kunyun Yang, A. Ertai, Shucai Wu, Shujun Geng, Zhihui Li, Mingwu Li, Yu Pang, Shenjie Tang

**Affiliations:** 1 Beijing Chest Hospital, Capital Medical University, Beijing Tuberculosis and Thoracic Tumor Research Institute, Beijing, 101149 China; 2 Souzhou 5th People’s Hospital, Souzhou, 215007 China; 3 Hunan Chest Hospital, Changsha, 410013 China; 4 Chest Hospital of Xinjiang Uygur Autonomous Region, Urumqi, 830049 China; 5 Hebei Chest Hospital, Shijiazhuang, 050041 China; 6 Kunming 3rd People’s Hospital, Kunming, 650041 China

## Abstract

We compared the positive rates of T-SPOT.TB and bacterial culture in the smear-negative PTB, and analyzed the factors affecting the results of negative T-SPOT.TB and bacterial culture. Retrospective evaluation of data from smear-negative PTB patients who underwent T-SPOT.TB and bacterial culture were done. The agreement and concordance were analyzed between T-SPOT.TB and bacterial culture. Multivariable logistic regression analysis was used to explore the factors associated with positive results of T-SPOT.TB and bacterial culture in smear-negative PTB. 858 eligible smear-negative PTB patients were included in the study. The agreement rate was 25.6% (22.7~28.5%) between T-SPOT.TB and bacterial culture in smear- negative PTB patients. The positive rate of T-SPOT.TB was higher than that of bacterial culture in smear-negative PTB patients (p < 0.001). There were nearly no concordance between T-SPOT.TB and bacterial culture (p > 0.05). Using multivariable logistic regression analysis we found that older age ≥ 60 years (OR = 0.469, 95% CI: 0.287–0.768) and decreased albumin (OR = 0.614, 95% CI: 0.380–0.992) were associated with negative diagnostic results of T-SPOT.TB in smear-negative PTB patients. Female (OR = 0.654, 95% CI: 0.431–0.992) were associated with negative diagnostic results of bacteria culture in smear-negative PTB patients. Our results indicated that the older age and decreased albumin were independently associated with negative T-SPOT.TB responses.

## Introduction

Tuberculosis remains a major global health problem. It causes ill-health among millions of people each year worldwide. According to the World Health Organization (WHO), the estimated global incidence of TB cases was 10.4 million in 2016^1^.

Despite recent advances in diagnostics, bacteriological diagnosis still plays an important role in diagnosing tuberculosis. One of the most common and simple methods for diagnosing pulmonary TB (PTB) worldwide is smear of acid-fast bacilli (sputum-smear microscopy)^2^. The sputum smear microscopy test was simple, but the sensitivity of the sputum smear microscopy test is relatively low^3^. In clinical practice, bacterial culture and T-SPOT.TB have been also used to diagnose PTB. The culture of Mycobacterium bacteria from sputum samples (sputum bacterial culture) is the current reference standard for diagnosis of tuberculosis. But it requires more developed laboratory capacity and takes a long time to provide a diagnostic result^1^. T-SPOT.TB (Oxford Immunotec, Abingdon, UK) is based on the *ex-vivo* overnight enzyme-linked immunospot (ELISPOT) assay^4^. T-SPOT.TB assay showed high diagnostic value in detecting active TB patients in low TB endemic countries in previous studies^5^.

China is a high-burden TB country, with an estimated incidence rate of 66/100,000 tuberculosis cases in 2015^2^. In China, PTB has generally been diagnosed by traditional methods that rely on clinical symptoms together with the results of bacteriology methods (including sputum smear microscopy and bacterial culture) and X-ray examinations, etc^6^. The T-SPOT.TB assay is also used for diagnosis of *M. tuberculos* is infection in China and provides evidence for the diagnosis of TB patients without microbiologic evidences^7,8^.

In particular, smear-negative PTB represents the majority of TB cases^9^. Yet, quick and accurate diagnosis of smear-negative PTB is a challenge to us^10^. Hence, clinicians have to turn to other diagnostic tools for laboratory evidences of smear-negative PTB^11,12^.

Although several previous studies have reported the performance of T-SPOT.TB in detecting pulmonary and extrapulmonary TB^13,14^, the data regarding the factors associated with positive T-SPOT.TB results among smear-negative tuberculosis patients is limited for the setting with high TB incidence. Hence, the aim of this study was to retrospectively compare the positive rate between T-SPOT.TB and solid culture in diagnosis of smear-negative PTB. In addition, we analyzed the factors affecting the results of T-SPOT.TB and bacterial culture in the diagnosis of the smear-negative PTB.

## Results

### Patient characteristics

A total of eligible 858 smear-negative PTB patients were included during the study period, and 68.1% of whom were male. Nearly a quarter was 60 years or older (22.8%). Around one fifth have a BMI less than 18.5 (20.3%). The retreated patients count for 16.7%. Other clinical characteristics of PTB patients are shown in Table 1.

**Table 1:** Clinical characteristics of smear-negative PTB patients (n = 858).

Clinical characteristics	n(%)
Male	584(68.1)
Age(>60years)	196(22.8)
BMI(<18.5)	174(20.3)
Contact of TB	51(5.9)
Vaccination of BCG	416(48.5)
Retreated	143(16.7)
Course of TB(<1 month)	236(27.5)
Diabetes	64(7.5)
Smoking	246(28.7)
Decreased Albumin	145(16.9)

BMI (Body Mass Index) is the weight in kilograms divided by the square of the height in meters.

Decreased Albumin: Albumin <30 g/L.

### The results of T-SPOT.TB and bacterial culture in smear -negative PTB patients

In T-SPOT.TB negative subgroup of 116 PTB patients, 99 (85.3%) were bacterial culture negative while 17 (14.7%) were bacterial culture positive. In T-SPOT.TB positive subgroup of 742 PTB patients, 621 (83.7%) were bacterial culture negative while 121 (16.3%) were bacterial culture positive.

In total, the positive rate of T-SPOT.TB was 86.5% (84.2~88.8%) in smear -negative PTB patients. The positive rate of bacterial culture was 16.1% (13.6~18.6%) in smear -negative PTB patients. The positive rates between the assays were compared with the McNemar test. The positive rate of T-SPOT.TB was higher than that of bacterial culture (P < 0.001).

### The concordances of T-SPOT.TB and bacterial culture in smear -negative PTB patients

The agreement rate was 25.6% (22.7~28.5%) between bacterial culture and T-SPOT.TB in smear-negative PTB patients. There was nearly no concordance between T-SPOT.TB and bacterial culture in smear-negative PTB patients (kappa = 0.005, P = 0.652). Inspection of the discordant pairs revealed that the bacterial culture was positive in 17 patients when the T-SPOT.TB test was negative and bacterial culture was negative in 621 patients when the T-SPOT.TB test was positive.

### The factors associated with positive results of T-SPOT.TB in smear-negative PTB patients

Multivariable logistic regression analysis showed that, after adjustment for gender, smoking and others factors, factors significantly associated with positive diagnostic results of T-SPOT.TB were age < 45years (OR = 2.132, P = 0.003), normal albumin (OR = 1.629,P = 0.046) (Table 2). That is, older age ≥ 60 years (OR = 0.469, 95% CI: 0.287–0.768) and decreased albumin (OR = 0.614, 95% CI: 0.380–0.992) were associated with negative diagnostic results of T-SPOT.TB. in smear-negative PTB patients.

**Table 2:** Factors associated with positive of the T-SPOT.TB diagnostic tests in smear-negative PTB patients.

Factors	Total N = 858	No.(%) with positive results	Univariate OR (95% CI)	Multivariate adjusted OR (95% CI)	P
Gender					
Male	584	508(87.0)	Reference		
Female	274	234(85.4)	0.875(0.579–1.323)		
Age					
<45years	469	425(90.6)	2.555(1.607–4.061)	2.132(1.302–3.489)	0.003
45–59years	193	162(83.9)	1.382(0.825–2.315)	1.287(0.763–2.171)	0.345
≥60years	196	155(79.1)	Reference	Reference	
Smoking					
No	612	526(85.9)	Reference		
Yes	246	216(87.8)	1.177(0.755–1.837)		
Diabetes					
No	794	687(86.5)	Reference		
Yes	64	55(85.9)	0.952(0.457–1.982)		
BMI					
<18.5	174	158(90.8)	Reference		
≥18.5	684	584(85.4)	0.591(0.339–1.031)		
Contact of TB					
No	907	694(86.0)	Reference		
Yes	51	48(94.1)	2.605(0.798–8.506)		
BCG					
No	442	371(83.9)	Reference		
Yes	416	351(84.3)	1.078(0.757–1.354)		
TB Category					
New	715	626(87.6)	Reference	Reference	0.211
Retreatment	143	116(81.1)	0.611(0.380–0.981)	0.730(0.446–1.195)	
Course					
<1 month	236	210(89.0)	Reference		
≥1month	622	532(85.5)	0.732(0.460–1.165)		
Albumin					
Decreased	145	114(78.6)	Reference	Reference	0.046
Normal	713	628(88.1)	2.009(1.272–3.173)	1.629(1.008–2.631)	

### The factors associated with positive results of the bacteria culture in smear-negative PTB patient

Multivariable logistic regression analysis showed that, after adjustment for age, smoking and others factors, factor significantly associated with positive diagnostic results of bacteria culture was male gender (OR = 1.529, P = 0.046) (Table 3). That is, female (OR = 0.654, 95% CI: 0.431–0.992) were associated with negative diagnostic results of bacteria culture in smear-negative PTB patients.

**Table 3:** Factors associated with positive of the bacteria culture diagnostic tests in smear-negative PTB patients.

Factors	Total N = 858	No.(%) with positive results	Univariate OR (95% CI)	Multivariate adjusted OR (95% CI)	P
Gender					
Female	274	34(12.4)	Reference	Reference	
Male	584	104(17.8)	1.529(1.008–2.321)	1.529(1.008–2.321)	0.046
Age					
<45years	469	85(18.1)	1.386(0.867–2.215)		
45–59years	193	26(13.5)	0.974(0.546–1.740)		
≥60years	196	27(13.8)	Reference		
Smoking					
No	612	102(16.7)	Reference		
Yes	246	36(14.6)	0.857(0.567–1.295)		
Diabetes					
No	794	126(15.9)	Reference		
Yes	64	12(18.8)	1.223(0.635–2.357)		
BMI					
<18.5	174	25(14.4)	Reference		
≥18.5	684	113(16.5)	1.179(0.738–1.886)		
Contact of TB					
No	907	131(16.2)	Reference		
Yes	51	7(13.7)	0.821(0.362–1.862)		
BCG					
No	442	67(15.2)	Reference		
Yes	416	71 (17.1)	1.152(0.800–1.658)		
TB Category					
New	715	114(15.9)	Reference		
Retreatment	143	24(16.8)	1.063(0.657–1.722)		
Course					
<1 month	236	30(12.7)	Reference		
≥1month	622	108(17.4)	1.443(0.933–2.230)		
Albumin					
Decreased	145	24(16.6)	Reference		
Normal	713	114(16.0)	0.960(0.593–1.553)		

## Discussion

Despite the substantial progress to combat TB, it remains a major global health problem in the world. The diagnosis and treatment of TB is important for tuberculosis control. However, the diagnosis of smear-negative PTB patients is perceived to be difficult. Our study mainly compared the positive rate and analyzed the factors affecting the results of T-SPOT.TB and bacterial culture in the smear-negative PTB patients.

In our study with a large number of samples, the agreement rate was 25.6% between T-SPOT.TB and culture in diagnosis of smear-negative PTB. About 3/4 of the diagnostic results were different, mainly among which the T-SPOT.TB test was positive while culture was negative in 621 patients.

The bacterial culture are the current reference standard for diagnosing PTB, but the positive rate of bacterial culture was low in diagnosing smear-negative PTB. Our study indicated that the positive rate of T-SPOT.TB was 86.5% (84.2~88.8%) in smear -negative PTB patients, higher than that of bacterial culture (16.1%, P < 0.001). One study showed that the positive rates of T-SPOT.TB in smear-negative PTB were above 80%^15^. If conventional tests such as smear and culture are negative, then a T-SPOT.TB result could improve diagnostic value. The T-SPOT.TB test should be used as a supplementary test to diagnose smear-negative PTB.

Our study revealed that the concordance between T-SPOT.TB and bacterial culture results was poor in smear -negative PTB patients. If the result of T-SPOT.TB was positive, only 16.3% bacterial culture was positive. If the result of T-SPOT.TB was negative, 85.3% bacterial culture was negative in smear-negative PTB. Although bacterial culture is a ‘gold standard’ diagnostic method, its’ sensitivity is relatively low in smear-negative PTB. T-SPOT.TB has high sensitivity and accessory diagnostic value in smear-negative PTB. The two methods complement each other, so as to improve diagnosis of smear-negative PTB and reduce misdiagnosis.

In our study, we identified male gender was significantly associated with positive results of bacterial culture in smear-negative PTB (OR = 1.529, 95 CI: 1.008–2.321), which accords with international trends that male were significantly more at risk of contracting and dying from TB than female^16^. Qadeer *et al*. found that the prevalence of bacteriologically positive TB was 1.5 times higher among men than among women in Pakistan^17^. Male were perceived to get TB more often than female, as they were more exposed to risk factors during both work and leisure time^18^.

We found that age ≥60 (OR = 0.469, 95% CI: 0.287–0.768) and decreased albumin (OR = 0.614, 95% CI: 0.380–0.992) were significantly associated with negative results of T-SPOT.TB in smear-negative PTB. This is consistent with a report that false negative results of TSPOT.TB are associated with older age^19^. Lee *et al*. found that advanced age and immunosuppression were independently associated with weak positive T-SPOT.TB responses^20^. A possible explanation of the higher negative rate of T-SPOT.TB in older is that they may be immunocompromised. Our study suggests that the results of T-SPOT.TB should be interpreted cautiously in elderly patients.

The sensitivity of interferon-gamma release assays (IGRAs) in the detection of Mycobacterium tuberculosis infection could be affected by the condition of immune systems^21^. For this reason, immune dysregulation can contribute to higher possibilities of negative results of IGRAs. A meta-analysis has showed that the pooled sensitivity of T-SPOT.TB in HIV patients was low (65%)^22^, which means the T-SPOT.TB as *ex-vivo* immunologic assay may relate with the immune status. We found that the positive rate of T-SPOT.TB was higher in smear-negative PTB with normal albumin than in smear-negative PTB with decreased albumin. The immune status of the albumin decreased PTB may be lower than that of albumin normal. One study in India found that serum albumin concentrations decreased as a risk factor for increased mortality in HIV-infected patients with tuberculosis^23^. If the immune status is low, the positive rate of the T-SPOT.TB maybe low too.

This study has some obvious limitations. First, all the smear-negative PTB patients enrolled in this study were inpatients rather than the outpatients. The patients’ conditions were poor. Hence, the positive rate of the two methods may overestimate the true positive rate. Second, Gene Xpert MTB/RIF assay has been endorsed by World Health Organization to improve the detection of bacteria-positive TB patients. Unfortunately, it was only approved for diagnosing rifampicin-resistant tuberculosis patients from smear-positive sputum samples by Chinese FDA during the study period. As a consequence, the absence of Gene Xpert MTB/RIF assay may result in the misclassification of study subjects. Despite these limitations, our results provide valuable insight to study the T-SPOT.TB and bacterial culture among smear-negative PTB patients.

In conclusion, our findings indicate that T-SPOT.TB and bacterial culture might provide different results when used in routine clinical practice in diagnosing smear-negative PTB. T-SPOT.TB has high sensitivity, and has more advantage over the bacterial culture in diagnosis of smear-negative PTB. The older age and decreased albumin were independently associated with negative T-SPOT.TB responses. Male were independently associated with positive bacterial culture.

## Methods

### Study subjects

**Figure 1:**
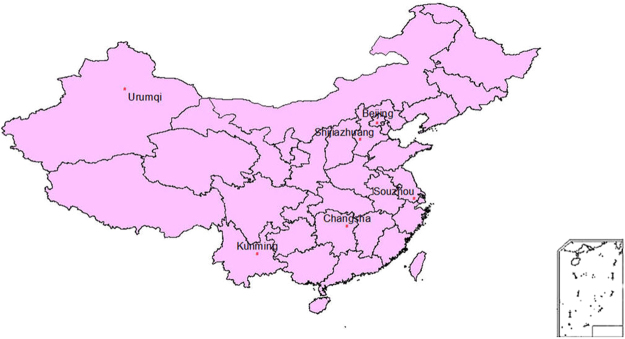
Distribution of smear-negative TB inpatients enrolled in this study.

We retrospectively recruited suspected PTB patient who were diagnosed at six Specialized Tuberculosis Hospitals in China between December 2013 and May 2015. PTB suspects were those who had at least one of the following symptoms: cough for >2 weeks, fever for >2 weeks, weight loss, TB contact history or radiological features. The definition of confirmed and clinically diagnosed PTB cases refers to the Clinical Diagnosis Standard of TB issued by WS 288–2008 in China^6^. The diagnostic criteria of sputum-negative TB in Chinese guideline include the following^6^: 1) clinical symptoms: cough for >2 weeks, fever for >2 weeks, weight loss, hemoptysis etc; 2) X-ray examination compatible with PTB; 3) bacteriological test negative; 4) anti-tuberculosis treatment effective; 5) other lung diseases excluded; 6) pathologic changes of tuberculous in lung; 7) tubercle bacillus found in bronchoalveolar lavage fluid; 8) PPD Test or IGRA positive; 9) TB antibody positive. The six hospitals were located in Beijing, Hebei Province, Xinjiang Province, Yunnan Province, Hunan Province, Jiangsu Province (Fig. 1). These hospitals represent eastern, central, and western regions of China, respectively. The incidence of PTB in the six provinces ranges from 32.0–184.5 /100,000 in 2015^24^.

The diagnosis of smear-negative PTB predominantly relied on clinical symptoms together with the results of bacterial culture, and X-ray examination and the effect of anti-tuberculosis treatment, etc. If smear-negative PTB inpatients had results of valid bacterial culture and T-SPOT.TB simultaneously, they were included in the study.

The results of the three diagnostic tests (sputum-smear microscopy, bacterial culture and T-SPOT.TB) were obtained from medical records. All patients showed negative serological findings for HIV.

This was an observational retrospective study and the three diagnostic tests were already used in clinical practice. Given that the medical information of patients was recorded necessarily and anonymously by case history, which would not bring any risk to the participants, the Ethics Committee of Beijing Chest Hospital, Capital medical university approved this retrospective study, with a waiver of informed consent from the patients.

### Clinical laboratory examinations

Three sputum samples were collected from each TB patient. Direct smears from each sputum specimen were examined with Auramine O staining for acid fast bacilli (AFB). The specimens were further digested with N-acetyl-L-cysteine and sodium hydroxide (NALC-NaOH) for 15 minutes. The alkaline solution was neutralized with phosphate buffer solution (PBS) to a total volume of 45 mL, and the suspension was centrifuged for 15 min at 3,000 × g. After the centrifugation, the supernatant was discarded and the sediment was re-suspended in 1.5 mL of PBS buffer. A 0.2 mL of suspension was inoculated onto the surface of Löwenstein-Jenson medium. The growth of mycobacteria on the L-J medium was checked per week after inoculation till the visible growth of mycobacterial clones. The culture was declared as negative if no growth on the L-J medium after 8-week incubation^6^.

The T-SPOT.TB assay was performed according to the manufacturer’s instructions using the T-SPOT.TB kit (Oxford Immunotec Ltd., Oxford, UK). Briefly, peripheral blood mononuclear cells were separated from the whole blood sample and incubated with the antigens (ESAT-6 and CFP10). The secreted cytokine by sensitized T cell was captured by specific antibodies on the membrane. Finally, the cytokine was detected by a chromogenic spot assay. Following manufacture instructions, the result of the testing was categorized “positive” or “negative” by s counting the number of spots^4^.

### Data management and statistical analysis

We took some measures to guarantee the data quality, including standardized study protocol and standardized training of research staffs. Trained health workers collected medical information by the use of a standardized questionnaire. From medical records we also obtained clinical characteristics of PTB patients such as age, gender, contact of TB, vaccination of BCG, albumin, body-mass index (BMI), smoking, etc. Qualitative data are presented as n (%).

Positive rate results across the two tests (T-SPOT.TB, bacterial culture) were compared using the McNemar test. The concordance between these two tests results were assessed using kappa coefficient. The statistic provides values of +1 (perfect agreement) via 0 (no agreement) to −1 (complete disagreement). Multivariable logistic regression analysis was used to determine which factors were associated with positive results of the TSPOT.TB and bacterial culture in smear-negative PTB. P < 0.05 was considered as statistically significant. All statistical analyses were conducted using SPSS software (version 13.0, Chicago, USA).
